# Development and implementation of a credentialing system for clinicians providing eating disorder care

**DOI:** 10.1186/s40337-025-01310-1

**Published:** 2025-07-17

**Authors:** Gabriella Heruc, Kim Hurst, Sarah Trobe, Beth Shelton, Emma Spiel, Siân A. McLean

**Affiliations:** 1 Australia & New Zealand Academy for Eating Disorders, Sydney, Australia; 2 Eating Disorders and Nutrition Research Group, Translational Health Research Institute, Western Sydney University, Sydney, Australia; 3 School of Applied Psychology, Griffith University, Gold Coast, Australia; 4 National Eating Disorders Collaboration, Melbourne, Australia; 5 Translational Health Research Institute, Western Sydney University, Sydney, Australia; 6 School of Psychology and Public Health, La Trobe University, Melbourne, Australia; 7 School of Medicine, Western Sydney University, Locked Bag 1797, Penrith, 2751 Australia

**Keywords:** Clinical practice, Clinicians, Credentialing, Eating disorders, Eating disorder treatment, Health professionals, Minimum standards, Professional recognition, Workforce development

## Abstract

With eating disorder prevalence increasing in Australia and around the world, it is critical that clinicians are trained in eating disorder treatment and that people with eating disorders, their families and supports can identify suitably trained clinicians. With funding from the Australian Government, the Australia & New Zealand Academy for Eating Disorders (ANZAED) and National Eating Disorders Collaboration partnered to develop and implement a novel credentialing system to recognise dietitians and mental health professionals with a minimum level of knowledge, experience, and training in eating disorder care. This paper aims to describe the development and implementation of this credentialing system, exploring its initial uptake by clinicians. In developing the Credential, initial scoping work included extensive consultation with clinicians, peak professional bodies, and those with lived experience of an eating disorder and their families. Once the criteria for the ANZAED Eating Disorder Credential were established and a custom website built, the Credential opened for applications from clinicians on 24 November 2021. To engage with clinicians, incentive schemes were used, including a limited evidence sunset period encouraging the existing workforce to apply, and professional development programs targeting the upskilling of clinicians new to eating disorder treatment. In the first 19 months of the Credential, 1410 clinicians (including 511 dietitians and 899 mental health professionals) were awarded the Credential. Although there was a small amount of anticipated attrition during the first renewal period, 1275 clinicians remained currently credentialed at 30 June 2023. Overall, most credentialed clinicians were private practitioners, likely motivated by the need for professional recognition. To support program improvements and long-term sustainability, future research will explore the benefits, impacts, and challenges of the credentialing system as perceived by clinicians and people with an eating disorder and their families.

**Supplementary Information:**

The online version contains supplementary material available at 10.1186/s40337-025-01310-1.

## Background

Eating disorders are serious mental health conditions that require skilled, evidence based care and treatment due to the complex physical risks and consequences, including high mortality rates from both suicide and physical complications [1, 2]. Internationally and in Australia, the prevalence of eating disorders has been steadily increasing over the last two decades, affecting individuals across all age groups and backgrounds [3, 4]. Approximately 70% of individuals impacted by eating disorders and associated issues are not linked with appropriate treatment providers [5]. Despite the growing need for access to effective treatment, only 20% of those who do receive treatment will receive eating disorder-specific treatment [5, 6]. Moreover, access to eating disorder treatment is impacted by a limited understanding [7] and lack of confidence [8, 9] in health professionals to provide evidence-based care for people with eating disorders, with 97% of clinicians reported to receive insufficient or no training in eating disorder management to enable them to confidently provide treatment [10]. Shortages of qualified professionals hinders timely, effective eating disorder treatment, leading to increased suffering and health risks [11]. Thus, to address the multifaceted challenges posed by these conditions, it is vital that there are adequately trained and experienced clinicians available to provide timely and appropriate treatment.

Recognising the urgency of addressing this issue, the Australian Government established an Eating Disorders Working Group through the Medicare Benefits Schedule (MBS) Review Taskforce in 2018 to develop recommendations aimed to ensure accessible, timely, and evidence-based treatment for individuals experiencing eating disorders [12]. The recommendations included expanding government funding for outpatient services, introducing subsidised support (through eating disorder-specific Medicare item numbers) for interprofessional collaborative stepped-care treatment of severe and complex eating disorders, and establishing a credentialing system for practitioners.

The focus of the present paper is on the process of development and implementation of an eating disorder treatment provider credentialing system for individual health care practitioners in Australia. Although financial sustainability of the system is a long-term goal, in light of the recent implementation of the system, sustainability forms a lesser focus in the present paper. In this context, credentialing refers to the formal process by which an individual health practitioner is recognised as meeting specified standards for practice in their particular discipline and typically involves ongoing requirements to maintain the credential [13, 14]. A license or registration with a health board or regulatory agency is a form of credential that may be required to practice. Credentialing may also be undertaken voluntarily to demonstrate acquisition of skill in a particular area of practice or for recognition of mastery or excellence [13, 15]. Health professionals in Australia are endorsed for their discipline specific practice by a range of boards or regulatory agencies. Due to the interprofessional nature of care for eating disorders whereby practitioners from medical, mental health, and dietetics contribute to treatment, general endorsement of discipline practice does not convey that health practitioners meet required standards in relation to eating disorders. Thus a specific credential that recognises relevant qualifications, knowledge, and training for provision of eating disorders treatment across all relevant health care disciplines is required to ensure appropriate standards are met. Moreover, overt display of credential status by practitioners is needed for individuals to be able to locate practitioners with the requisite training in evidence-based eating disorder treatment and ongoing skill development. It is suggested that a credentialing system in eating disorders may assist to address recognised difficulties in finding appropriately qualified health professionals [16]. These difficulties in navigating the healthcare system are frequently cited by people with eating disorders (e.g [17, 18]) and resultant delays in treatment or unsuitable treatment modalities can contribute to more entrenched disorders and poorer outcomes [19–21]. In response to the recommendation of the taskforce to establish a credentialing system, the National Eating Disorders Collaboration (NEDC), an initiative of the Australian Government Department of Health, commissioned a scoping report in 2019 to explore the feasibility and potential benefits of an eating disorder credential [21]. The report recommended that the Australia & New Zealand Academy for Eating Disorders (ANZAED), as the peak body representing professionals in the field of eating disorders in Australia and New Zealand, was the most suitable organisation to implement and manage this credentialing system. By establishing a credentialing system that (1) recognises clinician qualifications, knowledge, and training and (2) provides a publicly available searchable directory of credentialed clinicians, ANZAED and NEDC aimed to enhance the quality and consistency of care provided to individuals with eating disorders, ensuring that professionals across Australia are equipped with the expertise necessary to deliver evidence-based and person-centered care. Alongside this, ensuring visibility of credentialed clinicians through the database promotes referral and direct contact with clinicians, contributing to the second aim of the Credential to improve timely access to appropriate care. The present paper aims to describe the development and initial implementation period of the ANZAED Eating Disorder Credential.

### Development of a credentialing system for eating disorders

#### Co-design consultation process

**Fig. 1:**
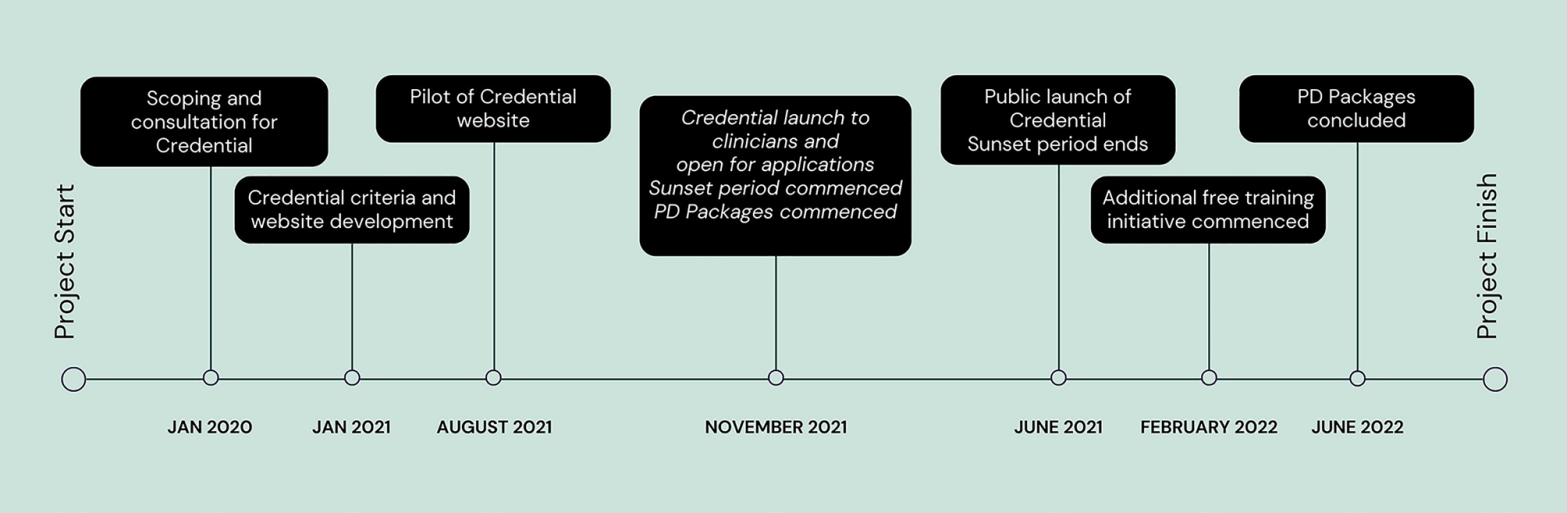
Timeline for the development and implementation of the Credential. Note. PD packages refer to the professional development incentives initiative

ANZAED and NEDC partnered to develop the ANZAED Eating Disorder Credential over three years (2020–2023) (see Fig. 1). The development phase involved extensive consultation with stakeholders, including individuals with lived experience, family members, health professionals, researchers, and relevant organisations, as well as an international literature review for similar initiatives.

The consultation process involved in-depth interviews, focus groups, a public workshop, and a national survey. Over 650 stakeholders nationwide participated, representing diverse perspectives. Full details of the participants involved and consultation process and findings can be viewed in the report published on the NEDC website [21]. Findings revealed strong support for a credentialing system whereby eligibility criteria reflect requirements for introductory level training, evidence-based practice, ongoing professional development, supervision, and professional body (or equivalent) registration. Stakeholders believed these components would enhance the quality of care and improve timely access to treatment for individuals with eating disorders [21]. Analysis of qualitative data collected in the consultation summarised the perceived benefits of credentialing with seven themes identified: protecting individuals with lived experience; empowering lived experience; enhancing professional care; safeguarding clinician equity; balancing safety, accessibility, and expertise; leveraging partnerships; and navigating risks.

### Development of the credential framework

Following these scoping activities, ANZAED and NEDC worked together to draft the eligibility criteria for the Credential. Several key guidelines documents provided the framework for developing the criteria, identifying eligible health professions, and informing training needs. These were the ANZAED Eating Disorder Treatment Principles and General Clinical Practice and Training Standards (referred to as ‘the Practice Standards’) [22], along with specific standards for mental health professionals [23] and dietitians [24], the NEDC Core Competencies [25], and desktop review of competencies covered within the tertiary education training of potential eligible health professionals that are currently involved in providing mental health treatment. Following extensive consultation with people with lived experience, carers, clinicians, professionals, service leaders, and other stakeholders, comprehensive criteria, eligibility parameters, and ongoing professional development requirements were established. This consultation involved 20 in-depth interviews, 2 face-to-face focus groups, a 100+ person public forum, a survey with 600+ responses, and 9 online focus groups. It was agreed that the Credential would initially be developed for dietitians and mental health professionals, with the latter including psychologists, mental health nurses, nurse practitioners, social workers, occupational therapists, counsellors, psychotherapists, psychiatrists and general practitioners (with additional mental health training). The endorsed criteria for the Credential are shown in Box 1. These include some minor differences in requirements for mental health professionals and dietitians, reflecting their different roles in eating disorder treatment and varying availability of evidence-based interventions for the professional roles.

**Table:** 

Box 1: The ANZAED Eating Disorder Credential criteria	
**Clinical experience**: • Two years’ mental health clinical practice (mental health professionals only). • Two years’ clinical dietetic practice (dietitians only). **Introductory training**: Introduction to eating disorders for health professionals training **Treatment provision training**: • Evidence-based treatment model for eating disorders (mental health professionals only)​ • Evidence-informed dietetic practice for eating disorders (dietitians only)​ **Ongoing annual professional development requirements**: • Supervision: 6 h per annum of supervision relevant to eating disorders (at least 3 h in 1:1 format)​ • Continuing professional development: 15 h per annum of CPD relevant to eating disorders​	

Ongoing annual supervision and professional development requirements (see Box 1) were established to ensure that clinicians continued to maintain and improve their clinical knowledge and skills. These requirements aligned with the profession-specific registration requirements for each professional group to ensure requirements were not overly burdensome. Clinicians could apply to be credentialed as a private practice provider, holding registration with their relevant professional body, and/or as an ‘other service provider’ with evidence of their clinical role provided by their employer for indemnity purposes.

#### Governance

Governance of the ANZAED Eating Disorder Credential has been managed by two separate processes. During the development phase, a Project Management Group was established with members from ANZAED and NEDC providing oversight and high-level decision making, through project planning and strategising. Following the public launch of the Credential (June 2022), oversight shifted to a Governing Council within ANZAED whose tasks include reviewing complex applications, monitoring operations, and guiding future development. Administrative processes, including credentialing application reviews, were integrated into a dedicated website, with a two-tier review system for quality control and dispute resolution.

#### Infrastructure support for the credential

Several mechanisms were required to be developed to support the operations of the Credential. Key mechanisms were a process to determine if training undertaken by applicants aligned with requirements of the Credential criteria and a website for Credential operations. In the absence of a pre-existing training course approval mechanism appropriate for the Credential, a training approvals process was developed by NEDC. Through a consultation process with key training providers across Australia (*N* = 15), scoping of needs of trainers and requirement for alignment of eating disorder training with national standards [22–24], competencies, and Credential criteria was conducted. A national Training Providers Reference Group was then established and, under NEDC’s leadership, the ‘National Framework for Eating Disorders Training (2021) – A Guide for Training Providers’ [26] was developed. This Framework, aligning with Credential requirements, outlined the guiding principles, general standards, and content standards necessary for inclusion in approved clinician training programs to become approved. Through the implementation of the Training Framework and Training Approvals process, consistency and quality in trainings required for the Credential would be achieved. Moreover, clinicians could identify trainings that align with the National Framework and agreed upon clinical standards.

In addition, to support the implementation and ongoing operations of the Credential, a website was developed to provide information on the Credential, to facilitate submission of clinician applications to become credentialed, and to provide a searchable directory through which credentialed clinicians could be identified for referrals. The website’s visual and functional elements were designed with a focus on accessibility, functionality, and professional imagery. Rigorous testing included automated and manual reviews, as well as user acceptability testing involving ANZAED, NEDC and lived experience representatives ensuring the website quality. A pilot evaluation of the usability of the website was conducted with clinicians and individuals with lived experience (*N* = 33). Feedback from testing led to further refinements to improve the overall user experience prior to public launch. Prior to launching the website to the public, lived experience perspectives (*N* = 18) of the revised website’s content and functionality were also gained through an online survey and an additional five representatives were involved in a roundtable discussion in May 2022. All lived experience representatives were recruited through not-for-profit organisations Butterfly Foundation, Eating Disorders Families Australia, Eating Disorders Victoria and Eating Disorders Queensland and InsideOut Institute.

During the development of the Credential, to foster awareness and interest in the Credential and to ensure ongoing stakeholder engagement, ANZAED and NEDC provided regular updates through member newsletters, social media platform posts, and targeted communications to relevant stakeholders across Australia, including peak professional bodies. The Project Management Group engaged in high-level meetings with professional governing and regulatory bodies, to discuss the rationale for credentialing, eligibility criteria, and governance aspects. These meetings were constructive, with governing professional bodies expressing interest and a willingness to engage in future dialogue. Stakeholder organisations also provided their logos for display on the credentialing website to endorse their support for the Credential.

#### Incentivising uptake

Several procedures were implemented to incentivise uptake of the Credential. The first was a time limited period of free applications during which applications would be processed for no fee. The second was the inclusion of a limited “sunset clause” whereby clinicians who did not have documentary evidence of having completed training (e.g., due to significant time lapse since training and varied formats for their learning) could apply for the Credential and provide testimonial account of equivalency of meeting the training criteria. The purpose of this clause was to ensure that experienced clinicians had a means of demonstrating that they met eligibility criteria and could be included in the Credential. These incentives were available in the initial period of the Credential from 24 November 2021 to 30 June 2022, prior to public launch.

The third incentive was provision of free professional development (PD) packages to support clinicians in meeting the training and supervision requirements for the ANZAED Eating Disorder Credential. Opening on 24 November 2021, interest in the packages was high, with 1,332 applications received from dietitians and mental health professionals to take part. Of these, 16 were ineligible due to being non-resident of Australia or not eligible to be credentialed. In addition, 199 clinicians withdrew prior to commencing. Reasons given were work pressures, change of role, lack of exposure to individuals with eating disorders, personal reasons, or parental or other leave. The packages were designed to support three different groups of clinicians based on their experience levels. Package 1, taken up by 378 mental health professionals and 141 dietitians, provided introductory training, treatment provision training, and supervision for clinicians new to treating eating disorders. Package 2, taken up by 136 mental health professionals and 88 dietitians: provided treatment provision training and supervision for clinicians with some experience in eating disorders. Package 3, taken up by 107 mental health professionals and 46 dietitians provided supervision support as required for the Credential and was for experienced clinicians who had already completed relevant training. Further information on uptake and characteristics of package recipients is in Additional file 1. A separate paper in this supplementary issue of the Journal of Eating Disorders [27] examines the impact from engagement with the professional development packages on clinicians’ knowledge, skill, and willingness to treat people with eating disorders, and explores organisational-level barriers to providing eating disorder treatment.

A final incentive program was offered after the initial PD package places were filled. In partnership with two online training providers, this initiative also offered free introductory and treatment provision training (but not supervision) to clinicians seeking to become credentialed who had not completed the training required to meet the criteria. To June 2023, 112 clinicians (65 mental health professionals and 47 dietitians) from every state and territory in Australia (see Supplementary Material 1 for distributions) took up these training opportunities.

#### Government funding investment

To support the development of the new credentialing system for eating disorders, the Australian Government Department of Health and Aged Care invested more than $2.5 million Australian Dollars (AUD) to cover the costs of resourcing and incentivising the program. Almost $1 million (AUD) of these funds directly supported the professional development and upskilling of the workforce to support more clinicians to meet the minimum standards of the Credential.

#### Launch of the credential

**Fig. 2:**
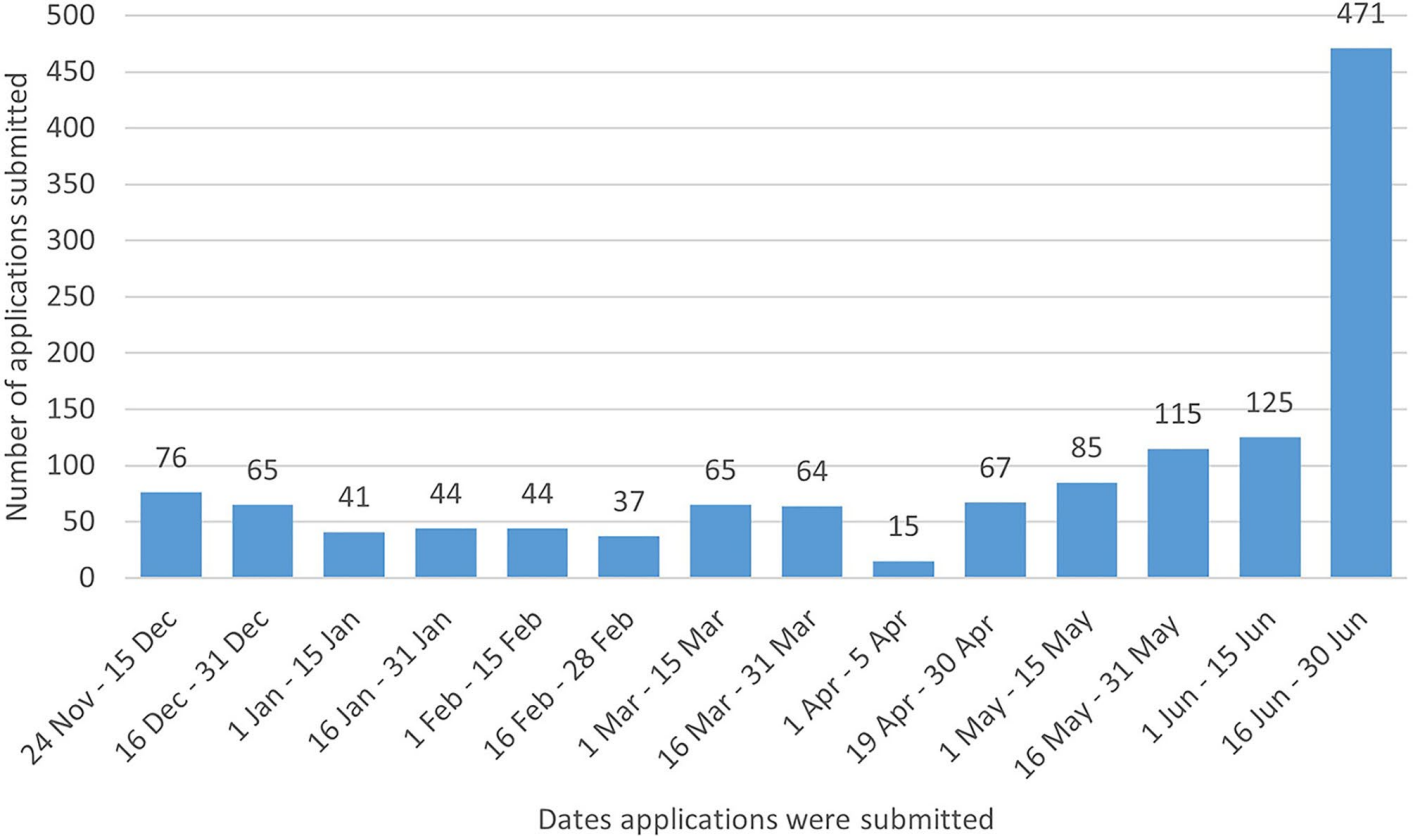
Number of sunset applications received fortnightly from 24 Nov 2021 to 30 Jun 2022. Note: the website was unavailable to the public between 6–18 April 2022

Applications for clinicians to become credentialed opened on 24 November 2021 in a targeted launch to health professionals in Australia. The initial sunset (and free application) period was available for six months, ending on 30 June 2022, during which time the bank of credentialed clinicians was built to ensure sufficient numbers prior to public launch. All clinicians applying during the initial period are referred to here as ‘sunset applicants’ and those applying from July 1, 2022 are referred to as ‘paid applicants’. Figure 2 shows the rate of applications across the “sunset” period. After being awarded the Credential, all sunset applicants were required to renew their Credential for the 1 July 2022–30 June 2023 period and pay the Annual Credential fee of $150 (AUD). From 1 July 2022, all new applicants (paid applicants) were required to pay a $100 administration fee plus the $150 Annual Credential fee (plus Australian Goods and Services Tax).

### Outcomes of the ANZAED Eating Disorder Credential

#### Applications and approvals

Uptake of the Credential by clinicians is explored here in relation to applications and renewals. The credentialing system received strong engagement following the opening of applications. By 30 June 2023, 1,767 completed Credential applications were received (1314 sunset and 453 paid applications), resulting in 1410 clinicians being awarded the Credential (1170 sunset applicants and 240 paid applicants post 1 July 2022 to 30 June 2023). As at 30 June 2023, 357 applications remained under review.

Table 1 shows the demographic and professional characteristics of clinicians awarded the Credential by location, profession, and sector. These data show that credentialed clinician reflect the characteristics of the Australian population, with most residing in the most populous states of Australia (New South Wales, Victoria, Queensland) and in metropolitan relative to regional and rural areas. Also reflecting discipline background of the eating disorder workforce in Australia, most credentialed clinicians are psychologists or dietitians, with smaller proportions having other mental health profession backgrounds. Furthermore, the majority of credentialed clinicians work in private practice settings.

**Table 1:** Breakdown of total clinicians awarded the Ccredential and currently credentialed by state/territory, geographical region, profession and practice setting

	Clinicians awarded the Credential (*n* = 1410)			Credentialed clinicians *not* renewed for 2022-23 (*n* = 139)	Currently credentialed clinicians^a^ (*n* = 1275)
	Sunset applicants (*n* = 1170)	Paid applicants (*n* = 240)	Total (% within category)	Total (% of sunset period applicants)	Total (% within category)
**Australian state/territory**^**b**^:					
Australian Capital Territory	30	7	37 (3%)	6 (20%)	32 (3%)
New South Wales	366	75	441 (31%)	44 (12%)	395 (31%)
Northern Territory	4	5	9 (< 1%)	0	8 (< 1%)
Queensland	255	52	307 (22%)	24 (9%)	285 (22%)
South Australia	58	8	66 (5%)	5 (9%)	59 (5%)
Tasmania	29	13	42 (3%)	2 (7%)	40 (3%)
Victoria	308	58	366 (26%)	41 (13%)	331 (26%)
Western Australia	120	22	142 (10%)	17 (14%)	125 (10%)
**Geographical area**:					
Metropolitan					1130 (89%)
Rural					138 (11%)
Remote					7 (< 1%)
**Profession**:					
Counsellor	29	7	36 (3%)	5 (17%)	31 (38%)
Dietitian	434	77	511 (36%)	29 (7%)	484 (38%)
General practitioner	8	1	9 (< 1%)	0	9 (< 1%)
Mental health nurse	29	5	34 (2%)	7 (24%)	27 (2%)
Nurse practitioner	3	2	5 (< 1%)	0	5 (< 1%)
Occupational therapist	19	2	21 (1%)	3 (16%)	18 (1%)
Psychiatrist	26	1	27 (2%)	5 (19%)	22 (2%)
Psychologist	558	124	682 (48%)	85 (15%)	599 (48%)
Psychotherapist	5	1	6 (< 1%)	0	6 (< 1%)
Social worker	59	20	79 (6%)	5 (8%)	74 (6%)
**Practice sector**:					
Private practice	688	154	842 (60%)	65 (6%)	796 (62%)
Other service provider^c^	257	50	307 (22%)	52 (4%)	251 (20%)
Both	225	36	261 (19%)	22 (2%)	228 (18%)

^a^ Currently Credentialed Eating Disorder Clinicians at 30 June 2023, including 1035 renewed clinicians during the 2022-23 period and 240 new applicants

^b^ Breakdown is reflective of Australian population distribution across states and territories, within +/- 2% for each state and territory (Australian Capital Territory = 1.7%; New South Wales = 31.2%; Northern Territory = 0.9%; Queensland = 20.5%; South Australia = 6.9%; Tasmania = 2.1%; Victoria = 25.7%; Western Australia = 10.9% [28])

^c^ ‘Other service provider’ includes all non-private practice clinical work settings

#### Renewals

During the first renewal period (1 July 2022- 30 June 23), 1019 renewals were processed and approved (of 1024 received). These were credentialed clinicians who had applied during the sunset period and renewed at the end of that period (30 June 2022). Of the originally approved 1,170 sunset applicants, 139 had not completed renewal of their Credential for the 2022-23 year or 2023-24 year (due to the timing of approval, 17 approved sunset applicants skipped 2022-23 and only renewed in 2023-24). Reasons for non-renewal were relocation (*n* = 1), non-response to request for further information needed to process the renewal (*n* = 5), renewals pending review and processing (*n* = 17), and unknown (*n* = 116). Non-renewing applicants were represented across most professions, with the highest proportion of non-renewals among mental health nurses (Table 1)). Non-renewing clinicians were from all states and territories except the Northern Territory with the highest rates amongst clinicians from the Australia Capital Territory (Table 1). In relation to practice sector, ’Other service providers’ had higher rates of non-renewal (20%) than those in the private practice sector only (9%) and those working in both private and other service provider sectors (8%).

During the second renewal period (1 July 2023–30 June 2024), a total of 940 renewals were received. These reflect renewals from the initial sunset applicant cohort who had renewed in 2022-23 as well as new applicants (original applicants approved 1 July 2022–30 June 2023) who were renewing for the first time in this 2023-24 period. At the time of reporting on 30 June 2023 (during the processing of the 2023–2024 renewal period), there were 1275 currently Credentialed Eating Disorder Clinicians (1035 renewed clinicians and 240 new applicants). It should be noted that renewals are allowed a 4-week grace period until 31 July for late renewal submissions.

#### Application processing

Although the program intended to process applications within 4 weeks, actual processing times varied, depending on the degree of complexity of the application and demand. For example, between January to April 2022, sunset applications were typically responded to within 4 weeks. From May to November 2022, response times for sunset applications slowed to an average of 45 days per application due to a surge in submissions immediately prior to the conclusion of the free application and sunset period (see Fig. 2). In May and June, application submissions increased by 55% and 201% compared to March, with more applications received in June (*n* = 601) than had been approved prior to that date. Moreover, for each application, professional registration/employment, and clinical experience and training needed to be assessed against the Credential’s criteria and the projected time for assessing sunset applications was underestimated initially. This was due to the unexpected length of applications (often exceeding 1,000 words for written evidence of training and experience), which may have contributed to longer times required for processing, ranging from 2 to 20 h per application. From May to September 2022, increased dedicated staffing time was required to assess these sunset applications (i.e. 2.0 full time equivalent (FTE)). In contrast, from 1 July, paid applications (providing evidence of completion of NEDC approved training, in lieu of lengthy written responses) were processed on average within 21 days, requiring just 1.0 FTE of staff time. Similarly, all 2022-23 and 2023-24 Credential renewal applications were reviewed within 4 weeks, taking on average only 3 days to process. At 30 June 2023, 524 (58%) of the 896 PD Package recipients were currently Credentialed Eating Disorder Clinicians.

#### Engagement with the credential

Since launching the ANZAED Eating Disorder Credential to the general public on 30 June 2022 through until 30 June 2023, the website developed specifically for the Credential [29] had received 191,925 pageviews, and 31,558 new users. This included 18,807 visits to the homepage, 16,550 visits to the ‘Find a Treatment Provider’ searchable directory page, 14,794 visits to the ‘my-account’ page, and 14,440 visits to the ‘apply and pay for the Credential’ page. Daily pageviews peaked at 2,220 on 4 April 2023, potentially coinciding with the highest number of newly credentialed clinicians with profiles on the ‘Find a Treatment Provider’ directory. Regarding the searchable directory, the majority of pageviews have occurred via direct links (120,078 views), with organic searches accounting for the second highest number of page views (38,921).

## Conclusions

The development of a credentialing system for eating disorders in Australia represents a novel initiative in workforce development and an important direction for improving pathways to evidence-based care for eating disorders. This development involved two years of consultation, planning, and design, followed by piloting and successful implementation, the latter of which was indicated by strong uptake by clinicians and public engagement with the searchable directory. With Australian Government funding and a strong partnership between ANZAED and NEDC, the ANZAED Eating Disorder Credential has been awarded to 1410 mental health professionals and dietitians from all Australian states and territories, with uptake across both the private and public sectors. Additionally, through the PD Packages and free training incentives, 1008 clinicians have upskilled in eating disorder treatment, contributing to essential workforce capacity.

Psychologists and dietitians represent the highest proportion of credentialed clinicians, reflecting their common roles in multidisciplinary care for people experiencing eating disorders. The smaller proportions of clinicians from other professions applying under the mental health professional pathway suggests an opportunity for future workforce capacity development to help address the growing need for eating disorder treatment. Social workers, occupational therapists, counsellors, psychotherapists, and mental health nurses with clinical mental health training and experience may be well-placed to upskill in evidence-based eating disorder care.

Importantly, clinicians from all states and territories of Australia have taken up the Credential, with representation from metropolitan, rural, and remote areas. Despite this, adequate provision of care in non-metropolitan areas is frequently a challenge and enhancing the credentialed workforce in rural and remote areas is needed. Several strategies such as task shifting, whereby care is shared within a team of providers [30], and use of telehealth for supervision and to build workforce capability [31], could further support clinicians based in rural and remote communities to take on the challenging work of providing eating disorder treatment.

Private practitioners were more strongly represented among those awarded the Credential than clinicians working in public health and other employed capacities as was expected from our initial scoping of relevance and motivations for a credentialing system. Australia’s shared public-private health system model, underpinned by a federally-funded Medicare rebate system, leads to eating disorder outpatient treatment frequently being delivered in the private sector. As the Credential has sought to ensure clinicians meet minimum clinical practice standards in eating disorder treatment, and with most evidence-based treatment (e.g. Cognitive Behavioural Therapy for Eating Disorders, Family Based Treatment for Eating Disorders, etc [32]). occurring in the longer-term outpatient setting, the Credential is particularly relevant to clinicians working in this sector in Australia. Moreover, private practitioners as small business owners are likely to be motivated to become credentialed for professional recognition and to promote themselves in the searchable directory to referrers and directly to those experiencing an eating disorder and in need of treatment. Indeed financial strain has been found to prospectively predict therapist turnover in publicly funded mental health settings [33] suggesting that financially relevant incentives, including indirect incentives in the form of increased referrals via the credentialing directory, are pertinent to maintain workforce capacity. Despite incentives appearing to be more relevant for private practitioners, 22% of clinicians awarded the Credential were other service providers (e.g. public health clinicians), with 80% of these opting to renew their Credential. This suggests that these clinicians were still motivated to renew, perhaps due to altruistically wanting to support people with lived experience having trust and confidence in their knowledge and training. Other countries considering a credentialing system may need to consider the relevant local drivers for clinicians, dependent on their health system models.

The credentialing system was successfully implemented after two years of development. A total of 1410 clinicians were awarded the Credential, while 1008 were upskilled through training programs. The credentialed clinicians represented a diverse range of professions and worked in both public and private sectors. The system has been successful and has made progress in the aim of enhancing workforce capacity to provide access to readily identifiable and timely care by a skilled workforce. Investigation of the impact of the Credential on delivery of care from the perspective of people with lived experience of eating disorders and carers is explored elsewhere in this supplement [34] and further understanding of the implications of the Credential on the system of care, such as improvements in timely access to treatment, would also be a useful future direction for research. Despite the success of credentialing to date, challenges in implementation were observed, including slower application processing than anticipated, non-renewals, and workforce gaps in certain professions. To address these issues, it is essential to streamline the application process, investigate reasons for non-renewal, and expand workforce development opportunities for underrepresented professions. Although NEDC’s initial consultation report identified some risks to developing a credentialing system (e.g. being a time and cost burden to clinicians, creating a barrier to service in rural areas, stopping eating disorder care being viewed as ‘core business’, not guaranteeing how “good” a clinician was) [21], further research exploring the reasons clinicians choose not to engage with the credentialing process may uncover unknown concerns or negative views about credentialing. By addressing these challenges, the credentialing system can continue to effectively enhance the eating disorder workforce and ensure long-term success.

While initial applications and uptake of the Credential by clinicians was critical to building the initiative, long term clinician retention is equally vital for sustainability and effectiveness. Of the initial 1410 clinicians awarded the Credential, 391 did not renew in 2022-23 and a further 79 did not renew in 2023-24. However, 240 new applications were received over that time, possibly reflecting workforce fluctuations and varying motivations for remaining credentialed.

Initially, the credentialing system aimed to become financially self-sustaining, with projections suggesting 1300 annual renewals were required to achieve sustainability. While this target has not yet been reached, strategies are underway to increase clinician retention and reduce operating costs through process automation. ANZAED is actively promoting the benefits of the Credential, targeting improving access to supervision and professional development, and creating incentives like free professional development webinars, online networking forums, and reduced ANZAED membership fees. New clinicians, including university graduates, are contributing to ongoing new applications, expanding the potential renewal pool. Incentives to compensate clinicians for the financial and time commitment to attain and retain the Credential are also needed as credentialing is an additional cost on top of compulsory profession-specific registration or membership fees. Recognition and promotion of utilisation of the searchable directory are crucial for this, especially for private practitioners. Ongoing refinements in the directory, search engine optimisation, and actions to increase public and referrer awareness aim to support this promotion.

The creation of the ANZAED Eating Disorder Credential is an innovative approach to connect individuals with eating disorders and their supports to mental health professionals and dietitians whose qualifications, training, and knowledge in providing safe and effective eating disorder treatment have been formally recognised. The credentialing program offers an incentive for clinicians to upskill in evidence-based care, and actively engage in ongoing professional development, addressing the need for a skilled eating disorder workforce. Ongoing efforts to enhance public awareness of the Credential will ensure help seekers can easily access the directory and identify Credentialed Eating Disorder Clinicians to increase the likelihood of people with an eating disorder finding the right care at the right time. Future long-term research examining the impact of support from a Credentialed Eating Disorder Clinician on treatment outcomes is essential to provide insights into the value and effectiveness of the Credential.

## Electronic supplementary material

Below is the link to the electronic supplementary material

Supplementary Material 1

## Data Availability

The datasets used and/or analysed during the current study are available from the corresponding author upon reasonable request. The data analysed and presented in this paper reflected a specific time-point in the operation of the credentialing system, with additional data unable to be provided due to both confidentiality and information technology constraints of data storage and access.

## Declarations

### About this supplement

This article has been published as part of Journal of Eating Disorders Volume 13 Supplement 1, 2025: The Australia & New Zealand Academy for Eating Disorders. The full contents of the supplement are available online at: https://jeatdisord.biomedcentral.com/articles/supplements/volume-13-supplement-1

## Abbreviations

ANZAED: Australia & New Zealand Academy for Eating Disorders
NEDC: National Eating Disorders Collaboration

## References

[1] Arcelus J, Mitchell AJ, Wales J, Nielsen S. Mortality rates in patients with anorexia nervosa and other eating disorders: a meta-analysis of 36 studies. Arch Gen Psychiatry. 2011;68(7):724–31. 21727255 10.1001/archgenpsychiatry.2011.74

[2] Smink FRE, van Hoeken D, Hoek HW. Epidemiology of eating disorders: incidence, prevalence and mortality rates. Curr Psychiatry Rep. 2012;14(4):406–14. 22644309 10.1007/s11920-012-0282-y PMC3409365

[3] Galmiche M, Déchelotte P, Lambert G, Tavolacci MP. Prevalence of eating disorders over the 2000–2018 period: a systematic literature review. Am J Clin Nutr. 2019;109(5):1402–13. 31051507 10.1093/ajcn/nqy342

[4] Hay P, Aouad P, Le A, Marks P, Maloney D, Barakat S, et al. Epidemiology of eating disorders: population, prevalence, disease burden and quality of life informing public policy in Australia—a rapid review. J Eat Disord. 2023;11(1):23. 36793104 10.1186/s40337-023-00738-7 PMC9933292

[5] Ali K, Radunz M, McLean SA, O’Shea A, Mavrangelos T, Fassnacht DB, et al. The unmet treatment need for eating disorders: what has changed in more than 10 years?? An updated systematic review and meta-analysis. Int J Eat Disord. 2025;58(1):46–65. 39482805 10.1002/eat.24306

[6] Striegel Weissman R, Rosselli F. Reducing the burden of suffering from eating disorders: unmet treatment needs, cost of illness, and the quest for cost-effectiveness. Behav Res Ther. 2017;88:49–64. 28110676 10.1016/j.brat.2016.09.006

[7] Walker S, Lloyd C. Issues experienced by service users with an eating disorder: a qualitative investigation. Int J Ther Rehabil. 2011;18(10):542–9.

[8] Denman E, Parker EK, Ashley MA, Harris DM, Halaki M, Flood V, et al. Understanding training needs in eating disorders of graduating and new graduate dietitians in australia: an online survey. J Eat Disord. 2021;9(1):27. 33602327 10.1186/s40337-021-00380-1 PMC7891015

[9] Lakeman R, McIntosh C. Perceived confidence, competence and training in evidence-based treatments for eating disorders: a survey of clinicians in an Australian regional health service. Australas Psychiatry. 2018;26(4):432–6. 29609472 10.1177/1039856218766124

[10] National Eating Disorders Collaboration. A nationally consistent approach to eating disorders: Opportunities to implement the National Eating Disorders Framework 2013.

[11] Kazdin AE, Fitzsimmons-Craft EE, Wilfley DE. Addressing critical gaps in the treatment of eating disorders. Int J Eat Disord. 2017;50(3):170–89. 28102908 10.1002/eat.22670 PMC6169314

[12] Eating Disorders Working Group. Medicare benefits schedule review taskforce: report from the eating disorders working group. Australian Government; 2018.

[13] Chappell KB, Howard MS, Lundmark V, Ivory C. Credentialing and certification: overview, science, and impact on policy, regulation, and practice. Int Nurs Rev. 2021;68(4):551–6. 34591976 10.1111/inr.12721

[14] Patel R, Sharma S. Credentialing. StatPearls. Treasure Island (FL): StatPearls Publishing; 2022. 30137789

[15] Hickey JV, Unruh LY, Newhouse RP, Koithan M, Johantgen M, Hughes RG, et al. Credentialing: the need for a National research agenda. Nurs Outlook. 2014;62(2):119–27. 24630680 10.1016/j.outlook.2013.10.011

[16] McLean SA, Hurst K, Smith H, Shelton B, Freeman J, Goldstein M et al. Credentialing for eating disorder clinicians: a pathway for implementation of clinical practice standards. J Eat Disord. 2020;8(62). 10.1186/s40337-020-00332-1 PMC7607662 33292654

[17] Johns G, Taylor B, John A, Tan J. Current eating disorder healthcare services - the perspectives and experiences of individuals with eating disorders, their families and health professionals: systematic review and thematic synthesis. BJPsych Open. 2019;5(4):e59. 31530301 10.1192/bjo.2019.48 PMC6646967

[18] Wilksch SM. Toward a more comprehensive Understanding and support of parents with a child experiencing an eating disorder. Int J Eat Disord. 2023;56(7):1275–85. 36942822 10.1002/eat.23938

[19] Andrés-Pepiñá S, Plana MT, Flamarique I, Romero S, Borràs R, Julià L, et al. Long-term outcome and psychiatric comorbidity of adolescent-onset anorexia nervosa. Clin Child Psychol Psychiatry. 2020;25(1):33–44. 30764636 10.1177/1359104519827629

[20] National Eating Disorders Collaboration. National eating disorders framework: an integrated response to complexity. Commonwealth Department of Health and Ageing; 2012.

[21] National Eating Disorders Collaboration. A credentialing system for eating disorder treatment in Australia. Consultation report Australia; 2020.

[22] Heruc G, Hurst K, Casey A, Fleming K, Freeman J, Fursland A, et al. ANZAED eating disorder treatment principles and general clinical practice and training standards. J Eat Disord. 2020;8(1):63. 33292546 10.1186/s40337-020-00341-0 PMC7653831

[23] Hurst K, Heruc G, Thornton C, Freeman J, Fursland A, Knight R, et al. ANZAED practice and training standards for mental health professionals providing eating disorder treatment. J Eat Disord. 2020;8:58. 33292542 10.1186/s40337-020-00333-0 PMC7604958

[24] Heruc G, Hart S, Stiles G, Fleming K, Casey A, Sutherland F, et al. ANZAED practice and training standards for dietitians providing eating disorder treatment. J Eat Disord. 2020;8:77. 33317617 10.1186/s40337-020-00334-z PMC7737344

[25] National Eating Disorders Collaboration. National practice standards for eating disorders. Australia: National Eating Disorders Collaboration; 2018.

[26] National Eating Disorders Collaboration. National framework for eating disorders training. Australia: Butterfly Foundation; 2021.

[27] Spiel EC, Barns R, Heruc GA, Hurst K, Trobe S, McLean SA. Outcomes of professional development to support capacity to provide eating disorder treatment and exploration of service level barriers. J Eat Disord. Submitted. 10.1186/s40337-025-01308-9 PMC12175328 40533813

[28] Australian Bureau of Statistics. National, state and territory population: Statistics about the population and components of change (births, deaths, migration) for Australia and its states and territories 2024 [Available from: https://www.abs.gov.au/statistics/people/population/national-state-and-territory-population/latest-release#states-and-territories

[29] connect.ed: Professionals credentialed in eating disorders. 2024 [Available from: https://connected.anzaed.org.au/

[30] Hoeft TJ, Fortney JC, Patel V, Unützer J. Task-Sharing approaches to improve mental health care in rural and other Low-Resource settings: A systematic review. J Rural Health. 2018;34(1):48–62. 28084667 10.1111/jrh.12229 PMC5509535

[31] Hurley J, Longbottom P, Bennett B, Yoxall J, Hutchinson M, Foley K-R, et al. Workforce strategies to address children’s mental health and behavioural needs in rural, regional and remote areas: A scoping review. Aust J Rural Health. 2024;32(3):462–74. 38572866 10.1111/ajr.13119

[32] National Institute for Health and Care Excellence. Eating disorders: recognition and treatment [NG69]. National Institute for Health and Care Excellence; 2017.

[33] Adams DR, Williams NJ, Becker-Haimes EM, Skriner L, Shaffer L, DeWitt K, et al. Therapist financial strain and turnover: interactions with system-level implementation of evidence-based practices. Adm Policy Mental Health Mental Health Serv Res. 2019;46(6):713–23. 10.1007/s10488-019-00949-8 PMC7083521 31203492

[34] McCormack M, Shand T, Conti J, Heruc G, McLean SA, Prnjak K et al. The perceived impact of the ANZAED Eating Disorder Credential: Perspectives of individuals with eating disorder lived experience and their carers J Eat Disord. Submitted. 10.1186/s40337-026-01690-y PMC13428425 42538565

