# Supplementary material for: Development and implementation of a credentialing system for clinicians providing eating disorder care
Source: J Eat Disord. 2025 Jul 17;13(Suppl 1):144. doi: 10.1186/s40337-025-01310-1 (PMC12272996; doi:10.1186/s40337-025-01310-1)
Supplement: Supplementary file 1 — Supplementary Material 1 [file 40337_2025_1310_MOESM1_ESM.docx]

### Credential Professional Development Packages (PD Packages)

To gain the ANZAED Eating Disorder Credential, clinicians are required to complete both introductory training and training in one evidence-based treatment model (mental health professionals) or in evidence-informed dietetic practice (dietitians), as well as having two years of general clinical practice experience. Once credentialed, clinicians must meet the ongoing professional development requirements of six hours of eating disorder-specific supervision (of which a minimum of 50% is in a 1:1 format) and 15 hours of continuing professional development (CPD) relevant to eating disorders per year.

While essential for maintaining the quality and value of the Credential, for some clinicians, the required training and supervision may be difficult to access. To support clinicians to meet the training and supervision criteria, the Credential Professional Development Packages (PD Packages) were designed to meet the needs of three broad clinician groups:

- **Package 1: Clinicians new to providing treatment for people living with an eating disorder:**

These clinicians require introductory training, training in one evidence-based treatment model (mental health professionals) or in evidence-informed dietetic practice (dietitians), and supervision.

- **Package 2: Clinicians with some experience in providing treatment for people living with an eating disorder**:

These clinicians have some introductory knowledge but lack specific treatment provision training and/or supervision. Clinicians will require supervision and training in an evidence-based treatment model (mental health professionals) or in evidence-informed dietetic practice (dietitians).

- **Package 3: Clinicians with experience in providing treatment for people living with an eating disorder:**

These clinicians have training in an evidence-based treatment model (mental health professionals) or in evidence-informed dietetic practice (dietitians), are already providing treatment for people living with eating disorder and may benefit from the opportunity of additional supervision to further enhance their practice. Clinicians will require supervision (without training).

The training opportunities available via the PD Packages align with the Credential criteria. These include:

- Introduction to eating disorders for health professionals
- Evidence-based treatment model (mental health professionals only)
  - Family Based Treatment (FBT)
- Cognitive Behaviour Therapy for Eating Disorders (CBT-E)
- Cognitive Behaviour Therapy – Guided Self Help (CBT-GSH)
- Specialist Supportive Clinical Management (SSCM)
- Evidence-informed dietetic practice (dietitians only)

Whilst other mental health treatment models are eligible under the Credential criteria, these four models (FBT, CBT-E, CBT-GSH, SSCM) were chosen as the most common treatment models used, those which training was more readily available, and models which provide a strong foundation for new clinicians to commence their work in this area.

All training providers under the PD Packages were required to have their training approved under the [NEDC Training Approvals](https://nedc.com.au/professional-development/credentialing/nedc-training-approvals/). Supervision packages include 6 hours of supervision, made up of three hours of individual supervision and three hours of group supervision.

#### Providers of training and supervision

In November 2021, NEDC sought to identify clinicians interested in being involved in the provision of the training and supervision activities under the PD Packages. **75 training and supervision providers across mental health and dietetics** were engaged to provide the PD Packages. See Figure 1 for breakdown of professional background of providers.


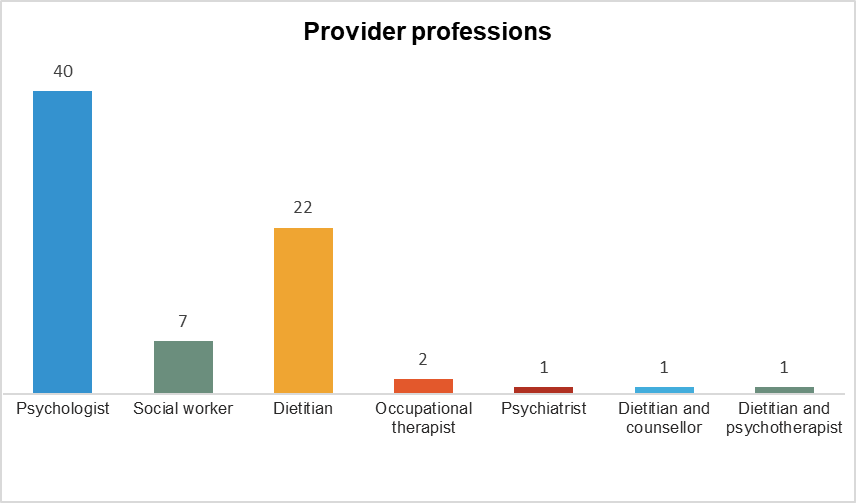


*Figure 1. Professional background of PD Package providers (supervisors and training providers) representing*

Training providers and supervisors were selected based on their level of experience in eating disorders, level of experience with a specific treatment model (mental health), and level of experience in training and/or supervision.

The providers were closely supported by NEDC staff with scheduling, communication with clinicians, marking attendance, providing resources to clinicians, and general administrative tasks.

#### Applications for the PD Packages

Applications for the PD Packages opened on 24 November 2021 and were made via the connect·ed website. Over the course of the program, NEDC received **1332 applications from mental health professionals and dietitians**, with 1095 applicants awarded a PD Package.

16 applications were not approved. Reasons for not approving included not being a resident of Australia, not being a profession eligible for the Credential, and/or not meeting the 2-year clinical experience criterion for the Credential.

Distribution of applicants across geographical location, discipline, and clinician experience is illustrated in combined figures below*.*

#### Participation in program

**896 clinicians engaged in the program**. ‘Engaged’ refers to completing some or all of the awarded training and/or supervision activities.

199 clinicians withdrew from the program prior to commencing their awarded training (P1 and P2) or supervision (P3). Reasons provided for their withdrawal included work pressures, change of role or workplace setting, not seeing people experiencing an eating disorder, personal reasons, and parental or other leave. Some clinicians have not provided a reason for their withdrawal.

Of the 896 clinicians that participated in the program, the allocation of PD Package type are as follows.

Table 1. Number of participants engaged in each PD Package type

| Package Type | Number of participants |
| --- | --- |
| Package 1 mental health | 378 |
| Package 1 dietitian | 141 |
| Package 2 mental health | 136 |
| Package 2 dietitian | 88 |
| TOTAL P1 and P2 | 743 |
| Package 3 mental health | 107 |
| Package 3 dietitian | 46 |
| TOTAL P3 | 153 |

The below figures illustrate geographical location, discipline, and clinician experience across applicants for and participants in the PD Package program.

*Figure 2. Percentage of PD Package applications (n=1332) and awarded PD Packages (n=896) by geographical area*

Based on [AIHW data](https://www.aihw.gov.au/reports/australias-health/profile-of-australias-population), rates of PD Package applicants and participants from regional, rural, and remote areas were higher than population rates. For participants, 37% of clinicians came from a regional, rural, or remote area compared to 27.8% of the population. These outcomes are in line with project aims, with targeted communications towards clinicians working outside of metro areas to ensure that this program helped with workforce shortages in these areas.

*Figure 3. Percentage of PD Package applicants (n=1332) and participants (n=896) by State and Territory against ABS population data.*

Applicants and participants broadly matched ABS population data. This aligns with project aims to ensure appropriate and representative engagement in the program from across Australia.


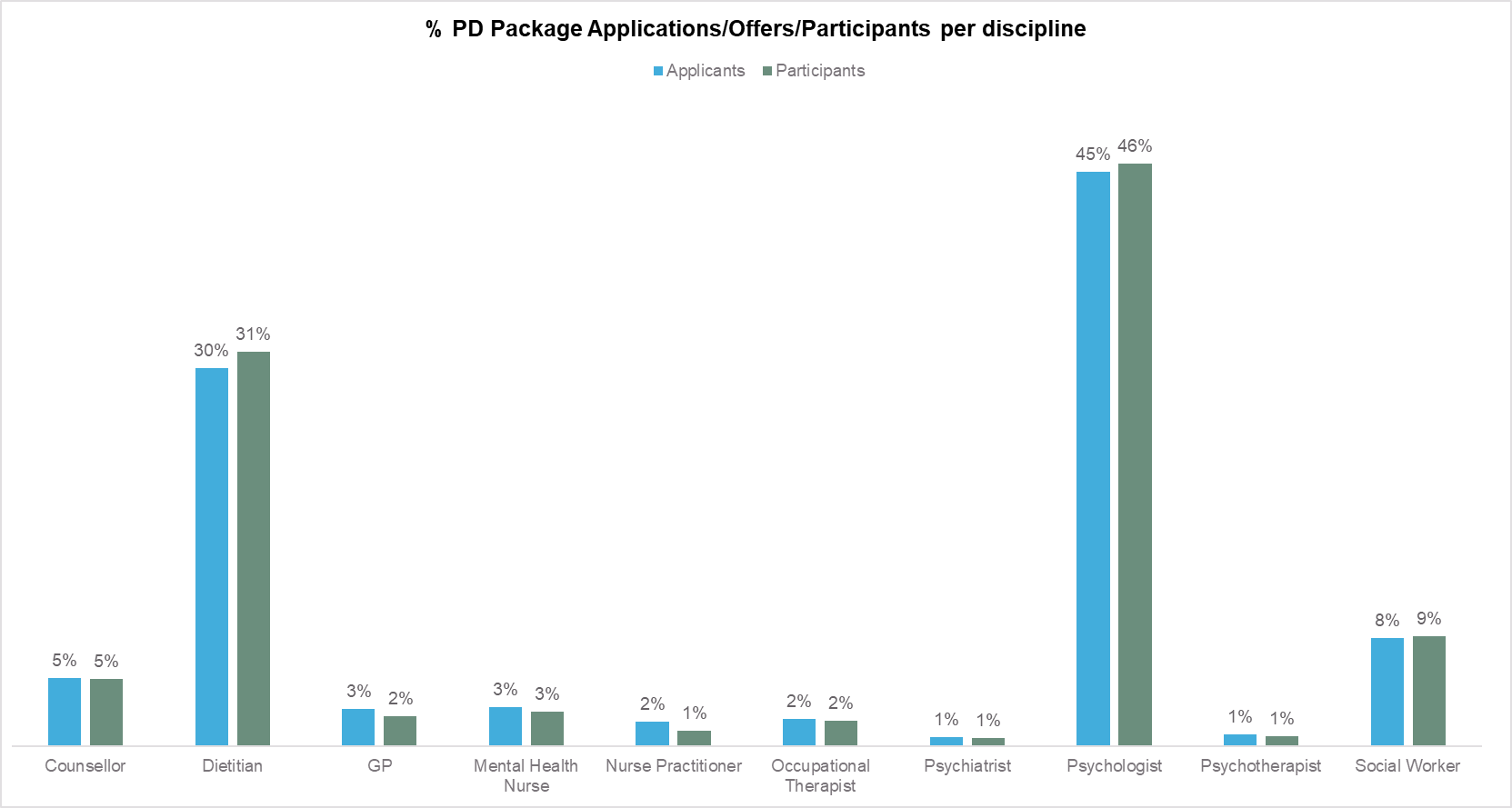


*Figure 4. Percentage of applicants (n=1332) and participants (n=896) by discipline*

Dietitians and psychologists made up the majority of applicants and participants in the PD Package program. Representation from all other professions eligible for the Credential was achieved (i.e., counsellors, GPs, mental health nurses, nurse practitioners, occupational therapists, psychiatrists, psychotherapists, social workers). The project planned for 30% of participants to be dietitians, with the remaining 70% to be mental health professionals to broadly align with workforce needs. Psychologists were predicted to make up the majority of mental health professionals, followed by social workers and occupational therapists. Applicant and participant data indicate that future workforce development initiatives would benefit from a focus on engaging a broader range of mental health professionals to enhance the size and diversity of the workforce that are ready, willing and able to provide evidence-based treatment for people experiencing eating disorders.

*Figure 5. Percentage of PD Package applicants and participants seeing people experiencing eating disorders prior to commencing their PD Package.*

Figure 5 indicates that whilst 84% of participants in the PD Package program were supporting people experiencing eating disorders prior to commencing their PD Package, only 21.75% of participants had completed treatment provision training (i.e., those awarded Package 3).

#### Training and supervision rollout

The PD Package trainings commenced on 28 February 2022 and extended until 24 March 2023. All trainings were provided online.

**Package 1 and 2**

Package 1 clinicians were awarded:

- Introduction to eating disorders training
- Evidence-based treatment model or evidence-informed dietetic practice training
- 3 hours of group supervision
- 3 hours of individual supervision

Package 2 clinicians were awarded:

- Evidence-based treatment model or evidence-informed dietetic practice training
- 3 hours of group supervision
- 3 hours of individual supervision

The full training calendar is as follows:

Table 2. Number of trainings provided under the PD Package program

| Training | Number of trainings |
| --- | --- |
| Online self-paced introduction to eating disorder training for mental health professionals | 1 |
| Combined Introduction to Eating Disorders + Dietetic Practice trainings | 8 |
| Cognitive-Behaviour Therapy – Enhanced (CBT-E) | 14 |
| Family Based Treatment (FBT) | 10 |
| Specialist Supportive Clinical Management | 2 |
| Cognitive Behaviour Therapy – Guided Self-Help | 4 |
| TOTAL | **39** |

Each training had attached supervision groups and individual supervision places available for each participant.

The program can be visualised in the following diagram:

Across the 38 live treatment provision trainings, 155 supervision groups and places for up to 810 clinicians were provided under Package 1 and 2. 67 clinicians enrolled in training but did not complete the full training or did not attend. Over the course of the program, 743 clinicians completed evidence-based treatment model training (mental health professionals) or evidence-informed dietetic practice training (dietitians). These clinicians are now eligible for the Credential. Breakdown of training type is illustrated below.

*Figure 6. Number of clinicians completing each type of training – CBT-Enhanced, Family Based Treatment, CBT-Guided Self-Help, Specialist Supportive Clinical Management, Evidence-Informed Dietetic Practice.*

CBT-E and FBT were requested more than SSCM and CBT-GSH. Trainings were scheduled based on clinician request and applicability to their workplace setting.

**Withdrawal before training**

160 clinicians withdrew from the PD Package prior to attending their training. Reasons provided related to work pressures, change of role or workplace setting, and personal reasons. Some clinicians have not provided a reason for their withdrawal.

**Attendance at training**

Across all 38 live trainings, the program achieved a 91% attendance rate. The breakdown across each type of training is shown in Table 4.

Table 3. Attendance rate at each trainings type run under the PD Package program

| Training | Attendance rate |
| --- | --- |
| Cognitive-Behaviour Therapy – Enhanced (CBT-E) | 92% |
| Family Based Treatment (FBT) | 92% |
| Specialist Supportive Clinical Management | 89% |
| Cognitive Behaviour Therapy – Guided Self-Help | 88% |
| Evidence-Informed Dietetic Practice | 95% |
| Average | **91%** |

**Attendance at supervision**

Group supervision: 155 groups were run, with 465 group supervision sessions (i.e., 3 sessions per group). Clinicians attended an average of 2.2 sessions. Dietitians attended an average of 2.6 sessions, with mental health professionals averaging 2.1 sessions per clinician. The breakdown of number of group supervision sessions attended is shown in Figure 7.


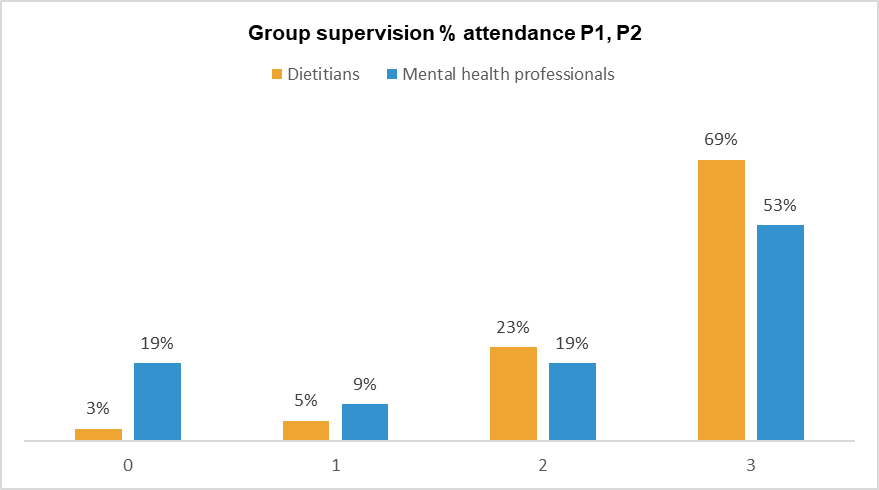


*Figure 7. Percentage of P1 and P2 mental health professionals and dietitians attending zero, one, two, or three sessions of group supervision.*

Individual supervision: Three individual supervision sessions were made available for each of the 743 Package 1 and 2 clinicians. Clinicians attended an average of 2.6 sessions. Dietitians attended 2.9 sessions on average, whereas mental health professionals attended 2.5 sessions per clinician. The breakdown of number of individual supervision sessions attended is shown in Figure 8.


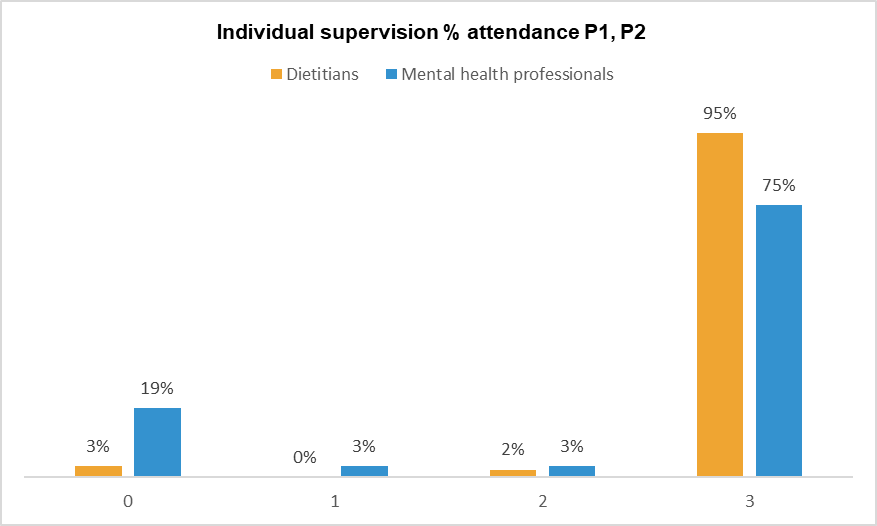


*Figure 8. Percentage of P1 and P2 mental health professionals and dietitians attending zero, one, two, or three sessions of individual supervision.*

**Package 3**

For those under the “experienced” PD Package pathway (Package 3), 32 supervision groups and places for 160 clinicians were available. Clinicians under the Package 3 pathway were credentialed and did not require training through the PD Package program. 7 clinicians withdrew from the program before accessing any of their supervision.

153 clinicians completed their group and individual supervision. These were 107 mental health professionals and 46 dietitians.

**Attendance at supervision**

Group supervision: Package 3 clinicians attended an average of 2.3 sessions. Dietitians were more likely to attend more sessions on average. Dietitians attended an average of 2.6 sessions, compared to 2.2 sessions for mental health professionals. The breakdown of number of group supervision sessions attended is shown in Figure 9.


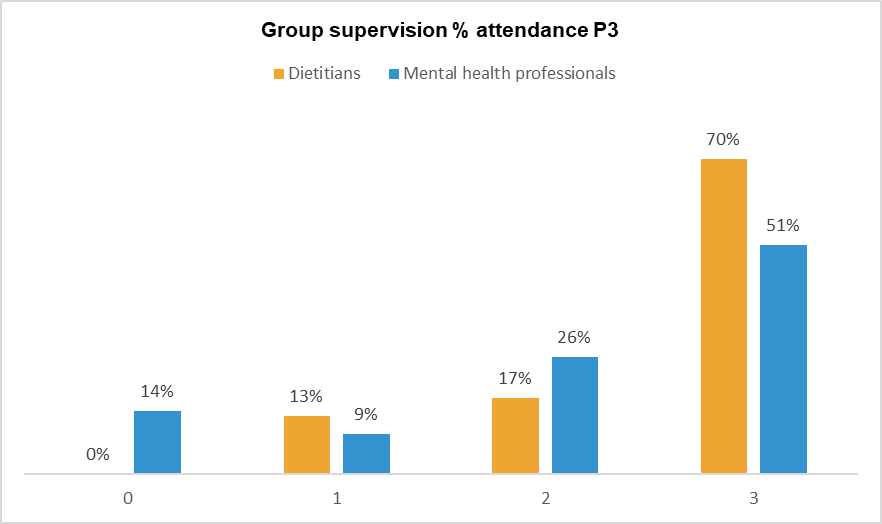


*Figure 9. Percentage of P3 mental health professionals and dietitians attending zero, one, two, or three sessions of group supervision.*

Individual supervision: Package 3 clinicians attended an average of 2.9 individual supervision sessions. Both mental health professionals and dietitians averaged 2.9 sessions per clinician. The breakdown of number of individual supervision sessions attended is below:


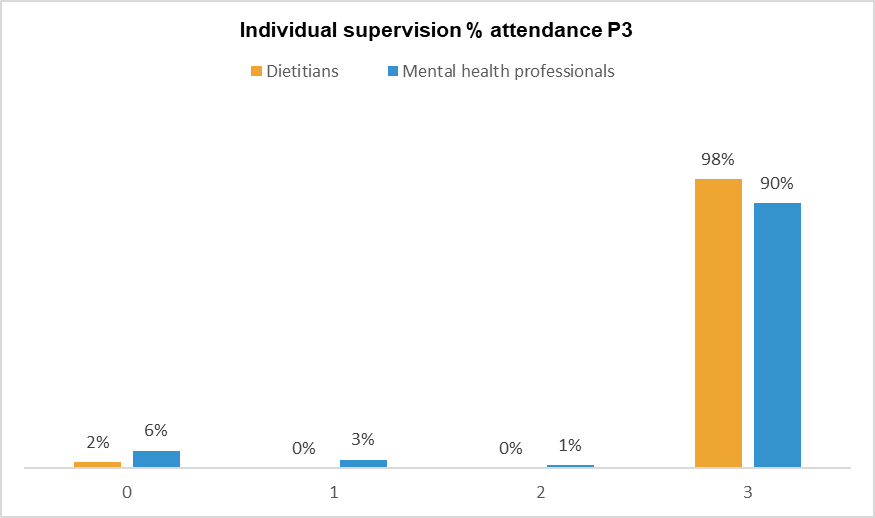


*Figure 10. Percentage of P3 mental health professionals and dietitians attending zero, one, two, or three sessions of individual supervision.*

Across all Package types, attendance at individual supervision was higher. Attendance at individual supervision was 89%, compared to 75% for group supervision. We can infer that the flexibility of scheduling individual supervision sessions at times most suitable for the clinician (as opposed to enrolling in a group that had scheduled dates and times) supported greater attendance.

**Summary of PD Package rollout**

The outcomes of the PD Package program met the aims of the project. It was aimed to support 600 clinicians to meet the eligibility criteria for the Credential. This project supported 896 clinicians to upskill in the area of eating disorders and become credentialed. The project also met aims across application and participation rates across each package type, geographical location of participants and professional groups.
